# Detection and characterisation of multi-drug resistance protein 1 (MRP-1) in human mitochondria

**DOI:** 10.1038/bjc.2012.40

**Published:** 2012-02-21

**Authors:** E A Roundhill, S A Burchill

**Affiliations:** 1 Children's Cancer Research Group, Leeds Institute of Molecular Medicine, St. James's University Hospital, Beckett Street, Leeds LS9 7TF, UK

**Keywords:** MRP-1, mitochondria, human, multi-drug resistance, Ewing's sarcoma family of tumours

## Abstract

**Background:**

Overexpression of plasma membrane multi-drug resistance protein 1 (MRP-1) can lead to multidrug resistance. In this study, we describe for the first time the expression of mitochondrial MRP-1 in untreated human normal and cancer cells and tissues.

**Methods:**

MRP-1 expression and subcellular localisation in normal and cancer cells and tissues was examined by differential centrifugation and western blotting, and immunofluorescence microscopy. Viable mitochondria were isolated and MRP-1 efflux activity measured using the calcein-AM functional assay. MRP-1 expression was increased using retroviral infection and specific overexpression confirmed by RNA array. Cell viability was determined by trypan blue exclusion and annexin V-propidium iodide labelling of cells.

**Results:**

MRP-1 was detected in the mitochondria of cancer and normal cells and tissues. The efflux activity of mitochondrial MRP-1 was more efficient (55–64%) than that of plasma membrane MRP-1 (11–22% *P*<0.001). Induced MRP-1 expression resulted in a preferential increase in mitochondrial MRP-1, suggesting selective targeting to this organelle. Treatment with a non-lethal concentration of doxorubicin (0.85 nM, 8 h) increased mitochondrial and plasma membrane MRP-1, increasing resistance to MRP-1 substrates. For the first time, we have identified MRP-1 with efflux activity in human mitochondria.

**Conclusion:**

Mitochondrial MRP-1 may be an exciting new therapeutic target where historically MRP-1 inhibitor strategies have limited clinical success.

Multi-drug resistance (MDR) contributes to a decrease in the efficacy of many agents used in the treatment of human malignancies. Overexpression of transmembrane proteins of the ATP-binding cassette (ABC) protein superfamily are commonly implicated in the development of MDR (Redmond *et al*, 2008). Multi-drug resistance protein 1 (MRP-1; (Cole *et al*, 1992)) is one of the first described ABC transporter proteins, expressed in the plasma membrane of normal and cancer cells where it effluxes both physiological toxins (Leier *et al*, 1994; Jedlitschky *et al*, 1997; Haimeur *et al*, 2004) and a variety of agents from the cell (Grant *et al*, 1994; Loe *et al*, 1998; Renes *et al*, 1999), leading to decreased cytotoxicity. In addition to being overexpressed in the plasma membrane of cancer cells, MRP-1 has been described in a variety of subcellular localisations, including the nucleus (Rajagopal and Simon, 2003), golgi apparatus (Kaufmann *et al*, 2008) and lysosomes (Rajagopal and Simon, 2003) of human cancer cells. Most recently, MRP-1 was described in the mitochondria of doxorubicin-treated cells of the murine sarcolemma (Jungsuwadee *et al*, 2009). Consequently, we have hypothesised that the subcellular localisation of MDR ABC transporter proteins may have a role in the detoxification of the cell by efflux of compounds from within specific subcellular compartments (Maraldi *et al*, 1999), which may modulate induction of the intracellular cell death cascade. To our knowledge, this is the first investigation to describe MRP-1 in mitochondria from human cancer and normal cells, and characterise its functional significance in a panel of human cell lines.

## Materials and methods

### Cell culture

The substrate adherent Ewing's Sarcoma Family of Tumour (ESFT; A673, RD-ES, SKES-1, SK-N-MC, TTC 466, TC-32) and the neuroblastoma (NB; SHEP-1 and SK-N-SH) cell lines were cultured as previously described (Myatt *et al*, 2005). The IMR-32 NB and HT-29 colon carcinoma cell lines were maintained in 50% Dulbecco's Modified Eagle Medium (DMEM; Invitrogen Life Technologies, Paisley, UK) and 50% RPMI (Invitrogen Life Technologies) containing 10% and 5% foetal calf serum (FCS; Harlan Sera-Lab, Leicestershire, UK), respectively, and 2 mM glutamine (Sigma-Aldrich, Dorset, UK). The transitional bladder carcinoma cell lines, RT-4 and RT-112 were cultured as previously described (Hurst *et al*, 2004). The breast adenocarcinoma (MCF-7), embryonal rhabdomyosarcoma (CCL136) and glioblastoma (T98G) cell lines were maintained in DMEM containing 10% FCS and 2 mM glutamine. The rhabdomyosarcoma cell line, A204, was cultured in McCoy's containing 10% FCS, 1.5 mM glutamine and 2.2g/l sodium bicarbonate (Invitrogen Life Technologies). The Phoenix A packaging cell line was used in the production of retroviruses (Tomlinson *et al*, 2005).

Normal human urothelial (NHU) cells were cultured as previously described (Chapman *et al*, 2006) and the normal human foreskin fibroblast (HFF) cell line was cultured in DMEM containing 10% FCS and 2 mM glutamine. Mesenchymal stem cells (MSC) were cultured in an undifferentiated state using nonhaematopoietic (NH) stem cell media; NH Expansion Medium (Miltenyl Biotec Ltd., Bisley, Surrey, UK).

All cell lines were purchased from the American Type Culture Collection (Manassas, Virginia, USA), except for the following cell lines which were gifts: RT-4, RT-112, NHU, T98G, HT-29, from Professor M Knowles (LIMM, SJUH, Leeds, UK), MSC from Dr E Jones (LIMM, SJUH, Leeds, UK), HFF from Cancer Research UK. All ESFT cell lines contain *EWS-ETS* gene re-arrangements and express CD99 at the cell membrane, characteristic of the ESFT. All cell lines are yeast, bacterial and mycoplasma-free; cells are evaluated for mycoplasma every 4 months using the EZ-PCR mycoplasma test according to manufacturer's instructions (Geneflow, Fradley, Staffordshire, UK).

### Subcellular localisation of MRP-1 by cellular fractionation and western blot

Subcellular fractionation (Westwood *et al*, 2002) and western blotting (Myatt *et al*, 2005) were performed as previously described. The purity of fractions was confirmed by western blotting for Grp75 (mitochondrial marker used at 1 : 1000; ab13529, Abcam Plc., Cambridge, UK) and NaKATPase (membrane marker used at 4 *μ*g ml^–1^; ab7671 Abcam Plc); equal loading was confirmed using the monoclonal *α*-tubulin antibody (0.4 *μ*g *μ*l^–1^; sc-5286 Santa-Cruz, CA, USA) or staining membranes for total protein using ponceau S (Sigma-Aldrich), where tubulin was not useful. MRP-1 protein expression was detected using the polyclonal antibody A23 (0.3 *μ*g ml^–1^; ALX-210-841 Axxora, Nottingham, UK) and Pgp expression using the monoclonal antibody C219 (1 *μ*g ml^–1^; Calbiochem, Merck Chemicals Ltd., Nottingham, UK). Western blots were incubated with primary antibodies overnight at 4^o^C and secondary antibodies (0.4 *μ*g ml^–1^; Alexa Fluor, Molecular Probes, Invitrogen Life Technologies, Invitrogen, Grand Island, NY, USA) for 1 h at room temperature with agitation. Bands were visualised and quantified using the Li-cor Odyssey infrared imaging system (Li-cor Biosciences, Lincoln, NE, USA).

### De-glycosylation of proteins

Subcellular fractions were prepared as previously described (Westwood *et al*, 2002) and proteins de-glycosylated using the Protein Deglycosylation Mix (New England Biolabs, Ipswich, MA, USA) following manufacturer's instructions for non-denaturing reaction conditions.

### Immunofluorescence (IF) and microscopy

Cells (2 × 10^4^) were grown on sterilised coverslips (22 × 40 mm; Scientific Laboratory Supplies Ltd., Hull, UK) in cell-specific media. MitoTrackerCMXRos (100 nM; M7512, Molecular Probes, Invitrogen Life Technologies), widely used to identify mitochondria (Zhou *et al*, 2011), was added to the cells for 30 min and cells then washed in PBS. If required, frozen primary tumour sections (5 *μ*m) were prepared. Sections and cells were fixed for 15 min at room temperature in 4% paraformaldehyde (in PBS; Sigma-Aldrich) and permeabilised with 0.1% Triton-X (BioRad, Hertfordshire, UK) for 5 min (in PBS; Oxoid, Nottingham, UK). Cells were incubated with primary antibody (5 *μ*g ml^–1^, A23 MRP-1 polyclonal antibody, Axxora; 1 : 200 Grp-75 (Zheng *et al*, 2008), ab13529, Abcam Plc; 1 : 100 *γ*-tubulin, ab11316, Abcam Plc.) for 1 h at room temperature, followed by a 30 min incubation with secondary antibody (0.4 *μ*g ml^–1^ Molecular Probes, Invitrogen, 100 *μ*l) also in PBS-containing DAPI (0.2 *μ*g ml^–1^, Sigma-Aldrich) at room temperature in the dark. In between incubations, sections and cells were washed in PBS. Cells were mounted using Dako Faramount Aqueous Mounting Medium Ready-to-use (Dako, Invitrogen Life Technologies, Stockport, UK). Sections were visualised by IF microscopy using a Zeiss 200 inverted microscope (Carl Zeiss Ltd., Hertfordshire, UK) and a Nikon Eclipse TE2000-E confocal microscope (Nikon UK Ltd., Surrey, UK). Z-stack images were rendered using the Nikon NIS-elements software; 0.2 *μ*m per stack. Control sections (no primary antibody) were included to check for cross-reactivity and bleed through of fluorescence. IF has been employed in these studies as by using the organelle-specific fluorescence marker MitoTrackerCMXRos it is possible to precisely visualise colocalisation of MRP-1 within the mitochondria.

### Isolation of functional mitochondria

Mitochondria were isolated from ESFT cells (12 × 10^7^) using the Mitochondria Isolation Kit (MITOISO2; Sigma-Aldrich) following the manufacturer's instructions. After isolation, mitochondria were re-suspended in 20 *μ*l of CellLytic M Cell Lysis Reagent (MITOISO2; Sigma-Aldrich) containing the Protease Inhibitor Cocktail (1 : 100; (v/v), MITOISO2; Sigma-Aldrich). For western blotting, the mitochondrial cell lysate was added to an equal volume of 2 × SDS reducing loading buffer containing 200 mM DTT. For functional assays, isolated mitochondria were resuspended in CellLytic M Cell Lysis Reagent, incubated with 1 *μ*M calcein-AM for 30 min and analysed by flow cytometry as described below.

### Activity of mitochondrial and membrane MRP-1

MRP-1-dependent efflux activity of mitochondria and whole cells was measured using the calcein-F efflux assay (Legrand *et al*, 1998). Briefly, whole cells were loaded with the non-fluorescent calcein-AM, which is converted to fluorescent calcein-F by intracellular esterases. Fluorescent calcein-F is then effluxed by MRP-1. Calcein-F-specific efflux by MRP-1 was confirmed using the inhibitor MK571: efflux of calcein-F was unaffected by the Pgp inhibitor verapamil (Supplementary Figure 1). ESFT cells were incubated for 30 min with calcein-AM; 0.05 *μ*M for whole cell or 1 *μ*M for isolated mitochondria analysis. Calcein-F accumulation and efflux in whole cells or mitochondria was measured on the FACsCalibur using an excitation laser of 488 nm and emission detected using a 530/30 nm filter (BD Biosciences, Oxford, UK). Unlabelled control samples were included to correct for autofluorescence.

### Knockdown of MRP-1 protein by siRNA

TC-32 cells were electroporated with MRP-1 siRNA (400 nM; siGenome SMARTpool M-007308-01-0005, Dharmacon, Lafayette, CO, USA) or scrambled siRNA control (400 nM; Silencer Negative control, Ambion, Austin, TX, USA) (Myatt and Burchill, 2008). MRP-1 protein expression, detected by western blot, was normalised to the loading control and relative to the scrambled siRNA control. MRP-1 efflux activity, after knockdown of MRP-1 protein by siRNA, was detected by measuring efflux of calcein-F (calcein-AM functional assay).

### Overexpression and subsequent characterisation of MRP-1 in the ESFT cell line TC-32

MRP-1 (a kind gift from Professor Cole; (Zhang *et al*, 2001)) was subcloned into the retroviral expression vector pFb-neo (Stratagene, La Jolla, CA, USA); the insert sequence was confirmed by terminator sequencing using the ABI PRISM Big dye terminator kit V1.1. and an ABI 3100 Genetic Analyser (Applied Biosystems, Invitrogen Life Technologies, GrandIsland, NY, USA) as previously described (Tomlinson *et al*, 2005). Infected cells were selected in geneticin (300 *μ*g ml^–1^; Sigma-Aldrich) for 10 days before placing cells in normal growth media. Overexpression of MRP-1 in the TC-32MRP-1.Fb-neo cells was confirmed by western blot and flow cytometry using A23 MRP-1 polyclonal antibody (Axxora). For flow cytometry (analysing 10 000 events) this antibody was used at 10 *μ*g ml^–1^ and expression detected using the goat-anti rabbit TRITC antibody (4 *μ*l ml^–1^; Southern Biotech, Birmingham, AL, USA). MRP-1 activity was evaluated by the calcein-AM functional assay.

Gene expression of 50 ABC transporters was measured using the Taqman Human ABC Transporter Array (Applied Biosystems, Invitrogen Life Technologies) according to manufacturer's instructions. RNA was extracted using the RNeasy Mini Kit (Qiagen, Crawley, West Sussex, UK) from TC-32.Fb-neo and TC-32MRP-1.Fb-neo cells. RNA (1 *μ*g) was converted to cDNA using Superscript III Reverse Transcriptase (Invitrogen) following manufacturer's instructions. Fold change in ABC transporter expression was determined using the comparative *c*_t_ method, normalising data to peptidylprolyl isomerase A (PPIA; Lastowska *et al*, 2007).

TC-32.Fb-neo and TC-32MRP-1.Fb-neo cells were treated with increasing concentrations of the MRP-1 substrates doxorubicin (0.85–56 nM), vincristine (2–120 nM), etoposide (7–240 nM) and a non MRP-1 substrate, actinomycin D (0.1–7.2 nM) for 48 h, after which viable cell number was counted using the trypan blue exclusion assay (Vi-cell, Beckman Coulter, High Wicombe, UK) (Myatt *et al*, 2005).

### Effect of chemotherapeutics on MRP-1 expression and efflux activity of ESFT cells

TC-32 cells were treated with doxorubicin (0.85 nM), etoposide (7.2 nM) or actinomycin D (0.1 nM) for 2–8 h. At these concentrations of the agents viable cell number was >80% (Figure 4B) and the concentration of actinomycin D was not sufficient to inhibit transcription (Cheon *et al*, 2000). From cell extracts subcellular fractions were prepared and MRP-1 expression analysed by western blot. Plasma membrane MRP-1 efflux activity was measured in both control and doxorubicin (0.85 nM) -treated cells using the calcein-AM functional assay.

The effect of the doxorubicin (0.85 nM) -induced increase in MRP-1 expression on the cytotoxicity of chemotherapeutics was examined in TC-32 cells. Briefly, doxorubicin (0.85 nM for 8 h) -treated and -untreated TC-32 cells were incubated with toxic concentrations of five chemotherapeutics (doxorubicin 3.5 nM, vincristine 8.6 nM, etoposide 61.8 nM, actinomycin D 0.2 nM and fenretinide 2.56 *μ*M) for 16 h, when an increase in MRP-1 was evident. Cells were harvested and apoptosis measured by flow cytometry of annexin V and propidium iodide (PI) -labelled cells (Annexin V-FITC apoptosis detection kit, BD PharMingen, BD Biosciences, Oxford, UK; Myatt *et al*, 2005); annexin V-PI was used to assess the cytotoxicity of agents as at 16 h cells may have an intact cell membrane (so would not be identified as dead with the trypan blue exclusion assay) but could be pre-apoptotic (annexin V positive) or apoptotic (annexin V:PI positive).

### Statistical methods

Unless stated otherwise, experiments were performed three times with triplicates in each experiment. Statistical analyses were performed using GraphPad prism (San Diego, CA, USA). Any significant differences in calcein-F efflux, MRP-1 expression, viable or non-viable cell number were determined using analysis of variance (ANOVA) followed by Bonferroni's *post hoc* multiple comparison test. Differences in gene expression were determined using ANOVA and Dunnett's *post hoc* test. Regression analysis was performed on viable cell counts to calculate the IC_50_ of therapeutic agents.

## Results

### Plasma membrane MRP-1 and its functional activity

**Figure 1:**
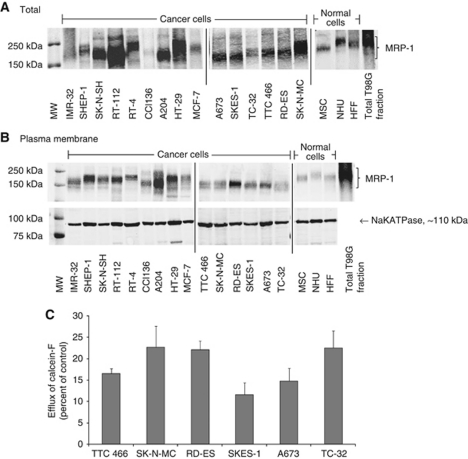
MRP-1 protein expression and activity in cancer and normal cell lines. Expression of MRP-1 in. (**A**) Total cell fraction and (**B**) plasma membrane fractions on western blot are shown. T98G cell extract is included on all blots as a positive control. TC-32 cell extracts were included on every original blot to allow comparisons between expression levels on different western blotting membranes. (**C**) The efflux of calcein-F from whole ESFT cells by plasma membrane MRP-1 following a pre-incubation with calcein-AM for 30 min. The results are expressed as percentage efflux of calcein-F compared with initial fluorescence following loading with calcein-AM. Results are shown as mean ±s.e.m. (*n*=9). Results of three independent experiments. Abbreviation: MW=molecular weight marker.

Full length MRP-1 (150–250 kDa) was expressed in all 15 cancer and 3 normal cell types examined (Figure 1A). The different sizes of native MRP-1 protein reflect post-translational glycosylation (Cole *et al*, 1992); de-glycosylated MRP-1 has a molecular weight of ∼150 kDa (Supplementary Figure 2).

Consistent with its role as a plasma membrane efflux protein, MRP-1 was detected in the membrane-enriched fractions of all the cells analysed (Figure 1B; Supplementary Figure 3). The SHEP-1, RT-112, A204, HT-29, RD-ES and A673 cancer cells had the highest membrane MRP-1 expression, whereas in cells of normal origin MRP-1 was barely detectable in the plasma membrane. This is consistent with the hypothesis that plasma membrane MRP-1 may be upregulated in some cancer cells. Consistent with its role as a transport protein, MRP-1-dependent efflux of calcein-F from ESFT cells was 12–23% over 60 min (Figure 1C). There was no statistically significant correlation between the level of MRP-1 in the plasma membrane (evaluated by densitometry of western blots) and efflux activity (*P*<0.1) in ESFT cells. However, when the expression of MRP-1 was decreased using MRP-1 siRNA in whole TC-32 cells (Supplementary Figure 4A), the efflux activity of MRP-1 was significantly decreased (*P*<0.05), (evaluated by the calcein-AM functional assay; Supplementary Figure 4B), supporting the use of the calcein-AM assay to evaluate MRP-1 efflux activity.

Consistent with the lack of Pgp protein expression in ESFT cells (Supplementary Figure 5A), no Pgp-dependent efflux activity was detected in ESFT cell lines (Supplementary Figure 1 and 5B).

### MRP-1 is expressed in the mitochondria of human cancer and normal cells

**Figure 2:**
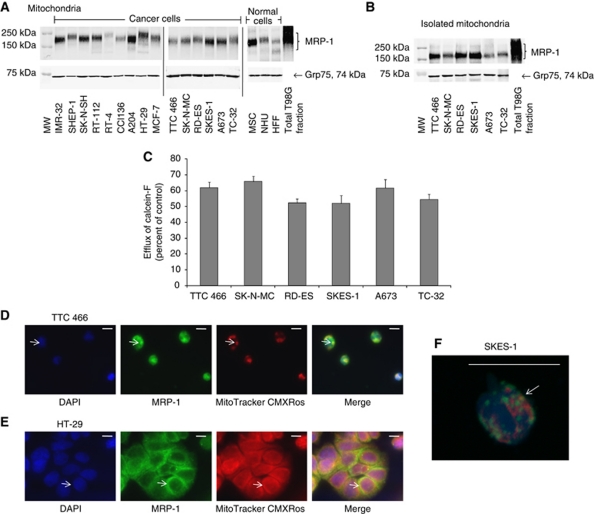
Mitochondrial MRP-1 expression and activity in cancer and normal cells. Expression of MRP-1 in (**A**) mitochondrial fractions on western blot are shown. T98G cell extract is included on all blots as a positive control. TC-32 cell extracts were included on every original blot to allow comparisons between expression levels on different western blotting membranes. (**B**) Expression of MRP-1 in the mitochondria isolated from ESFT cells. Equal loading was confirmed by expression of the mitochondrial marker Grp75. (**C**) The efflux of calcein-F from the mitochondria of ESFT cell lines following a pre-incubation with calcein-AM for 30 min. The results are expressed as percentage efflux of calcein-F compared with initial fluorescence following loading with calcein-AM. Results are shown as mean ±s.e.m. (*n*=9). Results of three independent experiments. (**D** and **E**) Co-localisation of MRP-1 and the mitochondrial-specific MitoTrackerCMXRos dye in cell lines. Cells have been labelled with MitoTrackerCMXRos (red; mitochondria), fixed and stained with DAPI (blue; nuclei) and MRP-1 (green). For each cell line the three different fluorescent stains and the merged image are shown; images are representative of each cell population analysed. Arrows indicate regions of co-localisation. Positive staining by multiple fluorescent compounds was compared with the staining with each individual compound alone to confirm the staining was not a result of interactions between the fluorescent compounds. (**F**) Z-stack images of SKES-1 cells created and rendered using the Nikon NIS-elements software. Scale bar=10 *μ*m. Abbreviation: MW= molecular weight marker.

MRP-1 was detected in the mitochondrial fraction of all cancer and normal cell lines (Figure 2A; Supplementary Figure 3). The level of expression was heterogeneous across the cell types examined. MRP-1 protein was more abundant in the mitochondrial protein mass in 7 out of 15 cancer cell lines (IMR-32, SK-N-SH, MCF-7, SK-N-MC, SKES-1, A673 and TC-32; Figures 1B and 2A, Supplementary Figure 3) than in the protein mass of the membrane fraction. In contrast, membrane MRP-1 protein was more abundant (Figure 1B) in 5 out of 11 (SHEP-1, RT-4, CCL136, A204 and RD-ES) cancer cell lines than mitochondrial MRP-1 protein (Figure 2A). Taken together, these data suggest there is subcellular-specific regulation of MRP-1 expression within the cell. Mitochondrial MRP-1 in normal cells was high in MSCs (Figure 2A), consistent with previous reports, suggesting that stem cells express high levels of ABC transport proteins (Zhou *et al*, 2001; Kim *et al*, 2002).

### MRP-1-dependent efflux from isolated mitochondria

Having observed mitochondrial MRP-1 in normal and cancer cell lines, it was important to evaluate the functional significance of the ABC transporter in this organelle.

The viability of the isolated mitochondria was confirmed by the successful oxidation of MitoTrackerCMXRos dye to a fluorescent form in isolated organelles measured by fluorescent microscopy (data not shown). The expression of MRP-1 was confirmed in isolated whole mitochondria from ESFT cell lines by western blot (Figure 2B). Efflux of calcein-F was observed in mitochondria isolated from all ESFT cell lines, consistent with mitochondrial MRP-1-dependent efflux activity; efflux of calcein-F was between 55-64% of the initial fluorescence within 5 min (Figure 2C) and calcein-F was completely removed from mitochondria by 10 min (cellular fluorescence was equal to the autofluorescence of unlabelled cells). The efflux activity from isolated mitochondria was greater than that from the whole cell via the plasma membrane (*P*<0.001). Interestingly, mitochondrial MRP-1 is glycosylated (Supplementary Figure 2), consistent with transport to the mitochondrial membrane and a putative efflux role.

### MRP-1 is expressed in the mitochondria of human cancer and normal tissues

Expression of MRP-1 in the mitochondria was confirmed by IF and microscopy using the mitochondrial marker MitoTrackerCMXRos. Expression of mitochondrial MRP-1 was either punctate (TTC 466; Figure 2D), suggesting cellular transport of MRP-1 in vesicles, or homogenous (HT-29; Figure 2E). Mitochondrial MRP-1 was also confirmed by analysing labelled cells by confocal microscopy (Figure 2F; Supplementary Figure 6).

This is the first time to our knowledge that MRP-1 has been described in mitochondria isolated from different human cell types that have not been exposed to chemotherapeutics. To confirm mitochondrial MRP-1 is not an *in vitro* phenomenon, we went on to investigate the expression of MRP-1 in the mitochondria of both normal and cancer tissues by IF and microscopy. MRP-1 was expressed in the membrane of all the tissues evaluated except the haemangioma tissue (data not shown).

**Figure 3:**
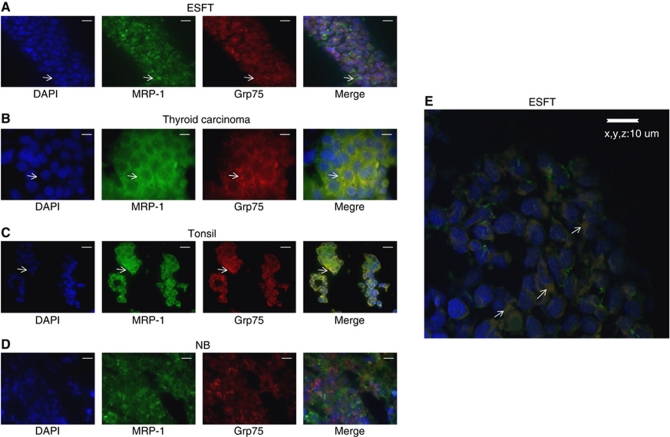
Co-localisation of MRP-1 expression and the mitochondrial-specific Grp75 in tissue sections. (**A**–**D**) Images of fixed tissue sections stained with DAPI (blue; nuclei), Grp75 (red; mitochondria) and for MRP-1 (green) are shown, in addition to a merged image. Images are representative of each tissue. Arrows indicate regions of co-localisation. Positive staining by multiple fluorescent compounds was compared with the staining with each individual compound alone, to confirm the staining was not a result of interactions between the fluorescent compounds. (**E**) Z-stack images created and rendered using the Nikon NIS-elements software. Images are representative of the tissue population analysed. Scale bar=10 *μ*m.

Consistent with the presence of mitochondrial MRP-1 in cancer cell lines, there was clear co-localisation of the mitochondrial marker Grp75 (red) and MRP-1 (green) in 7/7 primary ESFT (example shown in Figure 3A), 2/2 thyroid carcinomas (example shown in Figure 3B), 1/1 haemangioma, 2/2 melanomas and 1/1 soft tissue rhabdomyosarcoma. The expression of MRP-1 in mitochondria of primary tumours was confirmed by confocal microscopy (Figure 3E; Supplementary Figure 6). Consistent with the identification of mitochondrial MRP-1 in normal cells, co-localisation of Grp75 and MRP-1 was also observed in normal lymph node and tonsil (Figure 3C). However, MRP-1 was not evident in mitochondria of five NBs (example shown in Figure 3D), in contrast to the high mitochondrial MRP-1 observed in the NB cell lines (Figure 2A). Whether this reflects selection of NB cells surviving in culture conditions or an adaptation of cells to *in vitro* culture remains to be seen.

### Transport of MRP-1 to the mitochondria

**Figure 4:**
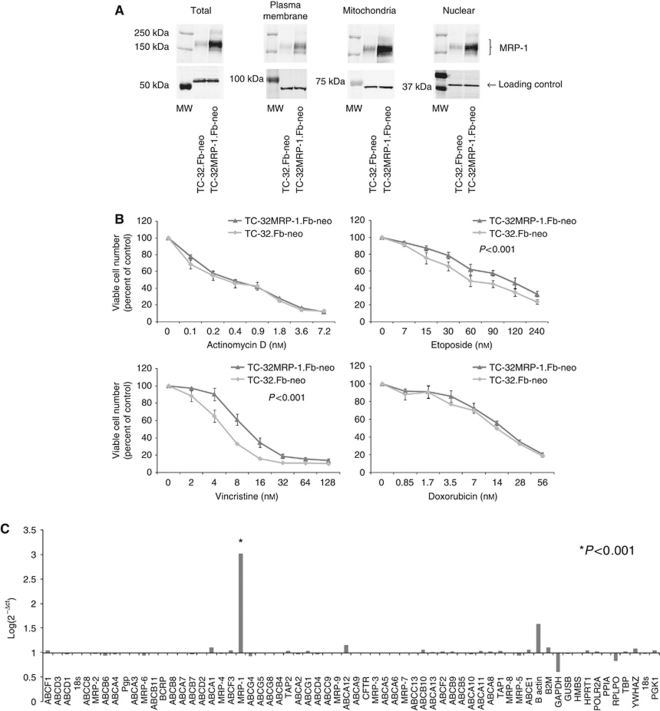
Expression of MRP-1 in TC-32MRP-1.Fb-neo cells. (**A**) Subcellular expression of MRP-1 in TC-32.Fb-neo control and TC-32MRP-1.Fb-neo cells. *α*-tubulin, sodium potassium ATPase, Grp75 and TATA TBP were included as loading controls for total, plasma membrane, mitochondrial and nuclear fractions, respectively. Results are representative of three independent sets of extracts. MW=molecular weight marker. (**B**) Percentage of viable TC-32MRP-1.Fb-neo and TC-32.Fb-neo cells, determined by the trypan blue exclusion assay, following incubation with doxorubicin (0.85–28), etoposide (7–240 nM), vincristine (2–60 nM) and actinomycin D (0.1–7.2 nM) for 48 h. Viable cell number expressed as mean percentage of vehicle control (±s.e.m.). Results of three independent experiments. (**C**) ABC transporter gene expression in TC-32.Fb-neo and TC-32MRP-1.Fb-neo cells. Gene expression calculated using the comparative *C*_t_ method, normalising expression to the endogenous control gene PPIA and expressed relative to ABC transporter expression in the TC-32.Fb-neo cells.

MRP-1 total protein expression on western blot was increased in the stable retroviral-infected TC-32MRP-1.Fb-neo cells, compared with the vector control-infected cells (TC-32.Fb-neo)(Figure 4A); this increase was approximately two-fold when quantified by flow cytometry (increase in fold change expression from 7±1 to 14±1; *P*<0.05) and correlated with an 85% increase in efflux activity measured using the calcein-AM assay (*P*<0.0001). Interestingly, expression of MRP-1 was preferentially increased in the mitochondria (densitometry, 634units±36; *P*<0.01) compared with expression in other subcellular localisations (Figure 4A, Supplementary Figure 8), suggesting that in conditions of overexpression MRP-1 is preferentially transported to the mitochondria in cancer cells. This may be analogous to the development of acquired resistance in cancer cells, although this requires further investigation.

Significant resistance to the cytotoxic agents vincristine and etoposide was induced in the TC-32MRP-1.Fb-neo cells compared with the vector control cells; IC_50_ of vincristine from 5 to 15 nM (*P*<0.001) and etoposide from 102 to 126 nM (*P*<0.001). There was no significant change in the IC_50_ for doxorubicin or actinomycin D (Figure 4B). Remarkably, MRP-1 was the only ABC transporter with significantly increased gene expression in the TC-32MRP-1.Fb-neo cells compared with the vector control (Figure 4C; *P*<0.001), consistent with the conclusion that the changes in sensitivity to vincristine and etoposide were likely to be the result of increased expression of MRP-1 rather than changes in the expression of other ABC transporters.

### Doxorubicin and etoposide increase the expression of MRP-1 in the membrane and mitochondria of TC-32 cells, inducing resistance to chemotherapeutics that are MRP-1 substrates

**Figure 5:**
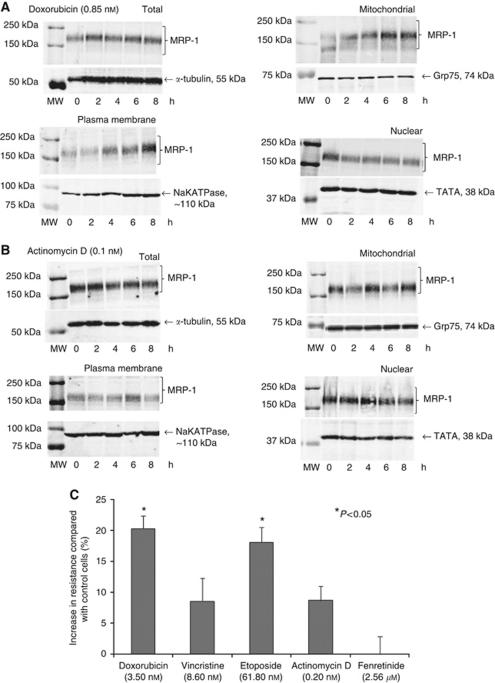
Subcellular expression of MRP-1 in TC-32 cells after incubation with (**A**) doxorubicin (0.85 nM) and (**B**) actinomycin D (0.1 nM). *α*-tubulin, sodium potassium ATPase, Grp75 and TATA TBP were included as loading controls for total, plasma membrane, mitochondrial and nuclear fractions, respectively. Results are representative of two independent sets of extracts. (**C**) Percentage increase in resistance to doxorubicin and etoposide after doxorubicin-dependent upregulation of mitochondrial and membrane MRP-1 expression. Percentage of viable, unlabelled TC-32 cells after pre-treatment with a non-toxic concentration of doxorubicin (0.85 nM) followed by a 16 h treatment with 3.5 nM doxorubicin, 8.6 nM vincristine, 61.8 nM etoposide, 0.2 nM actinomycin D and 2.56 *μ*M fenretinide, determined by annexin V/PI labelling of cells and flow cytometry. Results are shown as mean percentage increase in resistance±s.e.m. (*n*=9). Any significant differences in the percentage of unlabelled cells were determined by ANOVA and Bonferroni's *post hoc* test. Abbreviation: MW=molecular weight marker.

As we have established MRP-1 is expressed in the mitochondria of cancer cell lines, the expression of this MDR protein following exposure to chemotherapeutics was investigated to explore the hypothesis that mitochondrial MRP-1 might have a role in acquired drug resistance. Increased MRP-1 expression was not observed by western blot of total cellular fractions prepared from TC-32 cells treated with a non-toxic concentration of doxorubicin (Figure 5A). However, MRP-1 expression was increased in the membrane and mitochondrial fractions from these cells (Figure 5A). In agreement with these findings, efflux of calcein-F from the doxorubicin (0.85 nM, 8 h) -treated TC-32 cells was increased by 114±13% compared with the untreated control cells (*P*<0.01). In contrast, nuclear MRP-1 expression decreased 2 h after treatment (Figure 5A); this decrease was maintained to 72 h (data not shown). This may reflect the movement of MRP-1 from the nucleus to the mitochondria and plasma membrane. MRP-1 expression was not induced in the MRP-1-negative cytoplasmic fraction of TC-32 cells (data not shown).

Similar findings were observed when TC-32 cells were treated for up to 8 h with a second MRP-1 substrate, etoposide (Supplementary Figure 7). In contrast, treatment of TC-32 cells with the non-MRP-1 substrate actinomycin D did not increase expression of MRP-1 in total or specific subcellular fractions (Figure 5B). Interestingly, actinomycin D (a substrate of a second ABC transporter protein Pgp (Juliano and Ling, 1976)), did not induce Pgp expression in these cells (data not shown). Collectively, these observations demonstrate that chemotherapeutics that are substrates for MRP-1 (doxorubicin and etoposide) can upregulate expression of MRP-1 in specific subcellular organelles; this may be important for the development of drug resistance.

Consistent with this hypothesis, pre-treatment of TC-32 cells with doxorubicin (0.85 nM) significantly increased the resistance of this cell line to the cytotoxic effects of the MRP-1 substrates doxorubicin and etoposide after 16 h (*P*<0.05; Figure 5C). There was no significant increase in resistance observed after 16 h treatment with fenretinide or actinomycin D, consistent with the view that these agents are not substrates for MRP-1. Some agents such as vincristine need to be conjugated with a secondary factor such as glutathione for MRP-1-dependent transport (Loe *et al*, 1998), which may explain why there was no increased resistance to vincristine. Whether this is the case for fenretinide or actinomycin D remains to be seen.

## Discussion

In this study, we have demonstrated the expression of MRP-1 in the mitochondria of human cancer and normal cell lines and tissues. We found MRP-1 to be enriched in mitochondrial compared with plasma membrane protein extracts in a cell type-specific pattern. Although other ABC transporters have previously been identified in the mitochondria (Zutz *et al*, 2009) and more specifically MRP-1 has been described in mouse mitochondria isolated from heart tissue after treatment of mice with doxorubicin (11), this is the first time mitochondrial MRP-1 has been described in human, untreated cells. Importantly, we have shown mitochondrial MRP-1 has efflux activity, removing calcein-F more efficiently from viable mitochondria than from the whole cell. However, efflux activity does not correlate with plasma membrane MRP-1 expression. We are therefore currently investigating whether this may reflect the presence of functional MRP-1 splice variants and/or differences in ATP levels required by ABC transporters for function.

The punctate pattern of MRP-1 expression in the mitochondria is consistent with localisation to vesicles and a putative role in subcellular sequestration of toxins and protection of the organelle. Specific overexpression of MRP-1 in cancer cell lines increased resistance to chemotherapeutics that are MRP-1 substrates, and resulted in selective expression of MRP-1 in the mitochondria, suggesting this might have a role in acquired as well as intrinsic drug resistance. MRP-1 was also expressed in most cancer cell lines at higher levels in the plasma membrane than in normal cells, consistent with a putative role in the development of the malignant phenotype and drug resistance.

Efflux of calcein-F from isolated mitochondria is consistent with localisation of MRP1 to the mitochondrial membrane. This rapid and efficient efflux is similar to that of the Pgp substrate Rho123 from mitochondria of a drug-resistant hepatocellular carcinoma cell line (Solazzo *et al*, 2006). Some studies have suggested that mitochondrial Pgp is orientated in an inverse direction to that in the plasma membrane (Munteanu *et al*, 2006). However, the rapid efflux of mitochondrial calcein-F in this study confirms the orientation of mitochondrial MRP-1 to be the same as that in the plasma membrane, in agreement with Solazzo *et al.* (Solazzo *et al*, 2006). Indeed, calcein-F efflux from mitochondria was actually more efficient than that from the whole cells. Consistent with localisation to the mitochondrial membrane, mitochondrial MRP-1 is glycosylated; whether mutations in the putative glycosylation sites modulate expression and function as described for the Na^+^-dependent OCTN2 carnitine/organic cation transporter (Filippo *et al*, 2011) remains to be seen. These observations suggest that not only is mitochondrial MRP-1 likely to contribute to the development of the malignant and drug-resistant phenotype, it might also represent an exciting new therapeutic target.

Although the punctate pattern of mitochondrial MRP-1 expression is consistent with its presence and transport in vesicles, the mechanism of trafficking to the mitochondria is not clear. Previous studies investigating MRP-1 in the golgi of cancer cells suggest that localisation to this organelle may be due to a mutation or error in the MRP-1 transport pathway (Kaufmann *et al*, 2008). However, this is unlikely to be the explanation in these studies as overexpression of wild-type MRP-1 results in mitochondria-specific increased expression of the MRP-1 protein. Analysis of the MRP-1 sequence (using Predator and MitoProtII) failed to reveal published mitochondrial targeting sequences (Pfanner, 2000). We have therefore hypothesised that there is a currently unidentified mitochondrial targeting sequence in the NH_2_ terminus of MRP-1, and are investigating this possibility by site-directed mutagenesis (Neve and Ingelman-Sundberg, 2001). The increased expression of MRP-1 in the mitochondria following exposure to doxorubicin and etoposide suggests a tumour acquired resistant phenotype may be associated not only with increased expression of MRP-1 but with a specific increase in mitochondrial MRP-1.

The expression of MRP-1 in mitochondria of normal cell lines and tissues is consistent with recent data in the mouse identifying MRP-1 in mitochondria isolated from cardiomyocytes after treatment (Jungsuwadee *et al*, 2009). Consistent with our observations showing doxorubicin and etoposide increase mitochondrial MRP-1 expression, doxorubicin also increases the level of MRP-1 in the inner mitochondrial membrane of cardiomyocytes where it is reported to protect these cells from endo- and xeno-biotics (Jungsuwadee *et al*, 2009). Taken together, these studies demonstrate doxorubicin can increase mitochondrial MRP-1 in normal and cancer cells, and might therefore have a role in cellular homoeostasis and development of different abnormal phenotypes including the induction of multidrug resistance in cancer. The expression of MRP-1 in mitochondria isolated from MSCs is consistent with the role of the ABC transporters such as BCRP (Zhou *et al*, 2001) in stem cells, and also suggests increased mitochondrial MRP-1 expression may be important in the acquisition of cancer stem cell-like properties. We are currently investigating these possibilities.

Doxorubicin-induced expression of mitochondrial MRP-1 was accompanied by a decrease in nuclear expression, suggesting doxorubicin may affect intracellular trafficking of MRP-1. Exposure to doxorubicin significantly increased resistance to doxorubicin and etoposide but failed to increase resistance to the MRP-1 substrate vincristine, possibly reflecting the dependence of vincristine on GSH levels for MRP-1 efflux activity (Loe *et al*, 1996, 1998). However, specific retroviral-induced overexpression of MRP-1 triggered resistance to vincristine and etoposide. The difference in resistance observed following doxorubicin-induced increases in MRP-1 and overexpression using the retroviral expression vector most likely reflects chemotherapy-induced changes in drug resistance mechanisms other than increased expression of MRP-1 (Rajkumar and Yamuna, 2008). Therefore, our data suggest doxorubicin-induced resistance might reflect the increased efflux activity of mitochondrial MRP-1 protecting cells from induction of cell death. This hypothesis is supported by the co-localisation of MRP-1 and doxorubicin in lysosomes, suggesting the MDR protein directly transports doxorubicin into intracellular vesicles (Rajagopal and Simon, 2003).

In summary, we have identified mitochondrial MRP-1 in human normal and cancer cell lines and tissues, and confirmed mitochondrial MRP-1 efflux activity in human cell lines. In this subcellular organelle, MRP-1 may efflux chemotherapeutics and cellular toxins to protect the mitochondrial DNA from damage, and prevent induction of mitochondria-dependent cell death. It might therefore be considered a pro-survival protein, essential for normal cellular function and development of intrinsic and acquired drug resistance in cancer cells. Although other mechanisms of drug resistance have been described, given the key role that mitochondria have in oncogenesis and the process of malignant cellular transformation (Ralph *et al*, 2010) and the limited clinical success with conventional MDR inhibitors (Gandhi *et al*, 2007; Lhomme *et al*, 2008; Ruff *et al*, 2009), mitochondrial MRP-1 may represent an important new therapeutic target.
