# Supplementary figures and images for: Detection and characterisation of multi-drug resistance protein 1 (MRP-1) in human mitochondria
Source: Br J Cancer. 2012 Feb 21;106(6):1224–33. doi: 10.1038/bjc.2012.40 (PMC3304412; doi:10.1038/bjc.2012.40)

## Slide 1
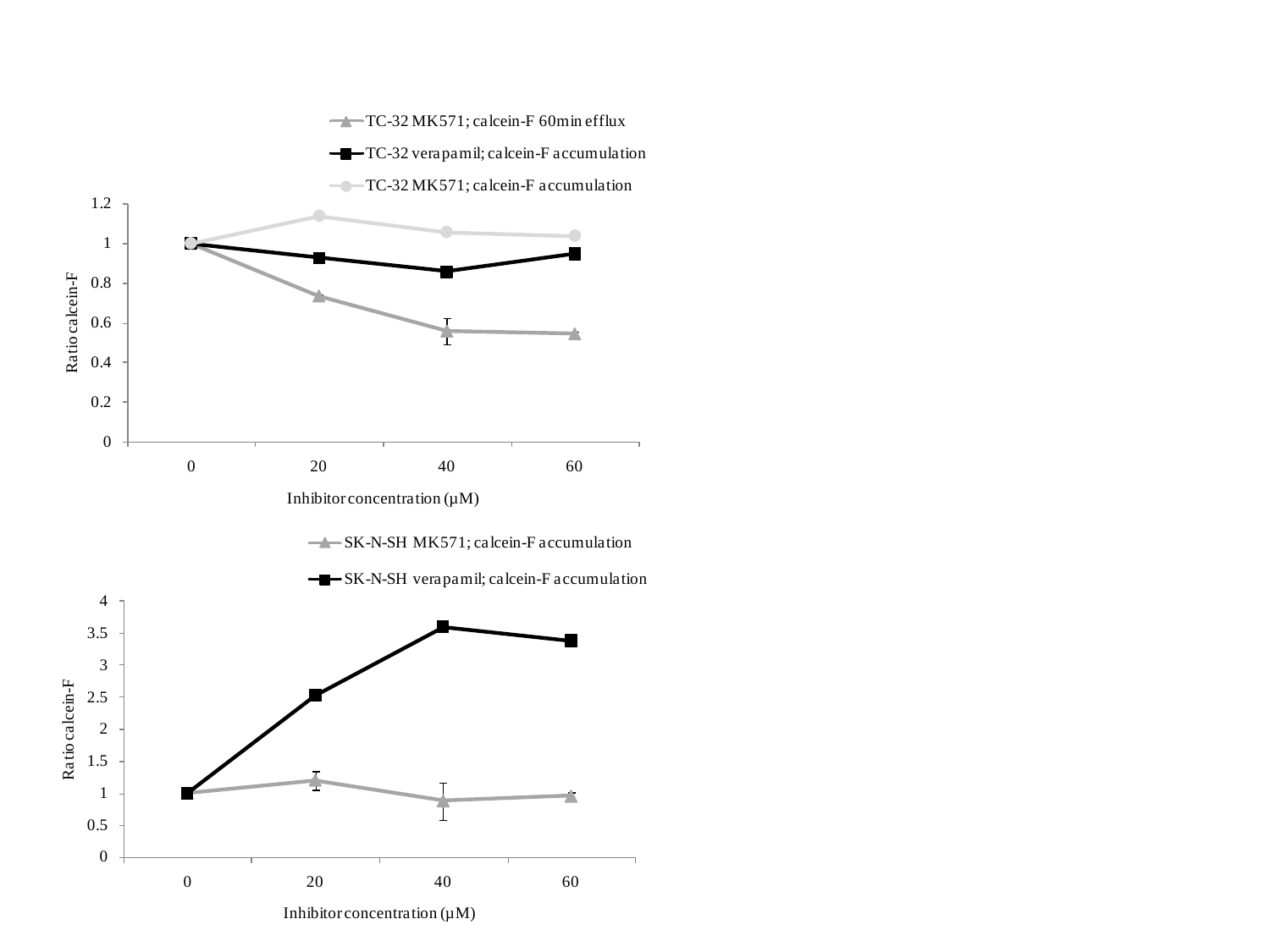

Supplement: Supplementary Figure 1 [file bjc201240x1.ppt]

## Slide 1
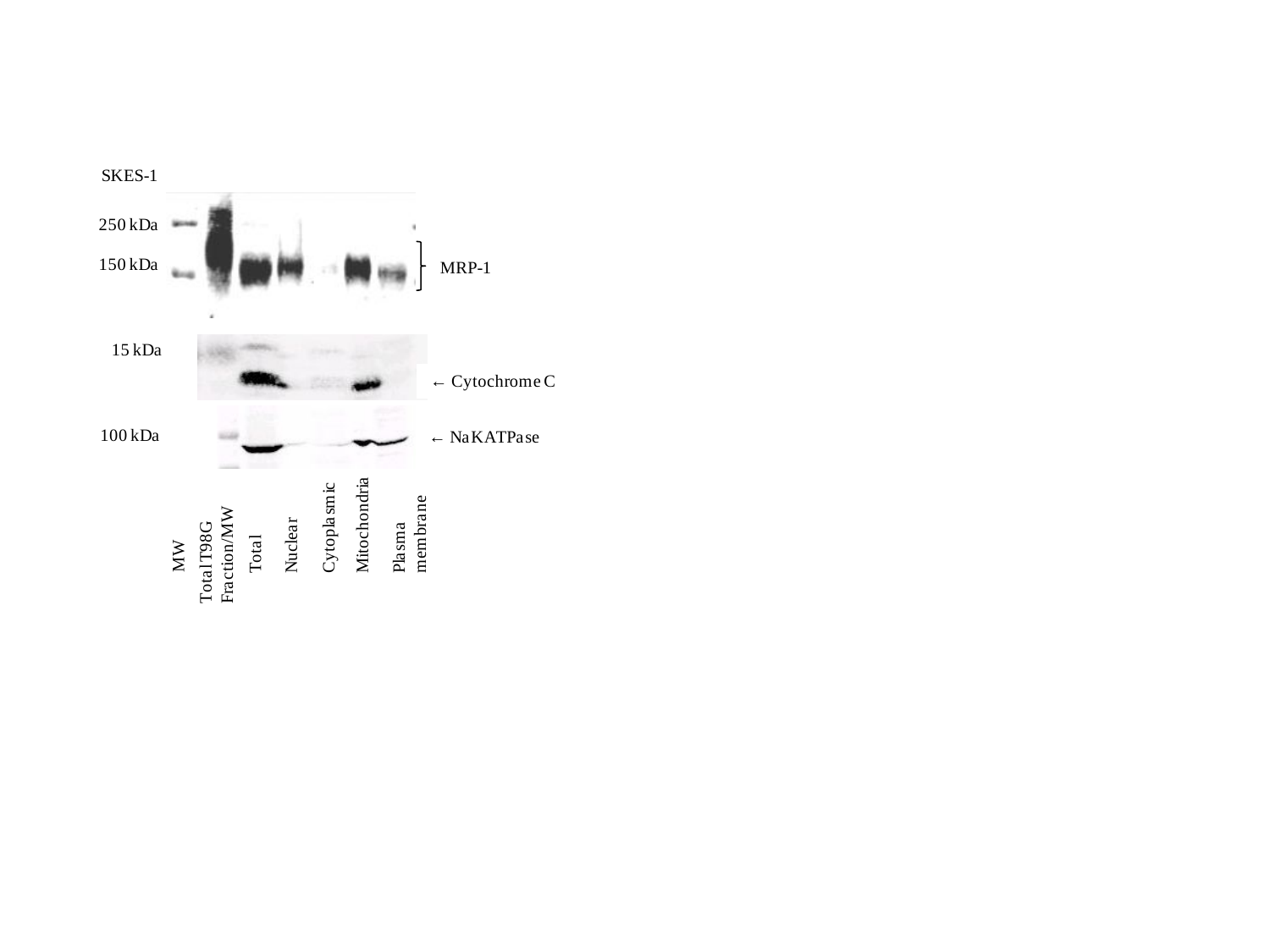

Supplement: Supplementary Figure 3 [file bjc201240x3.ppt]

## Slide 1
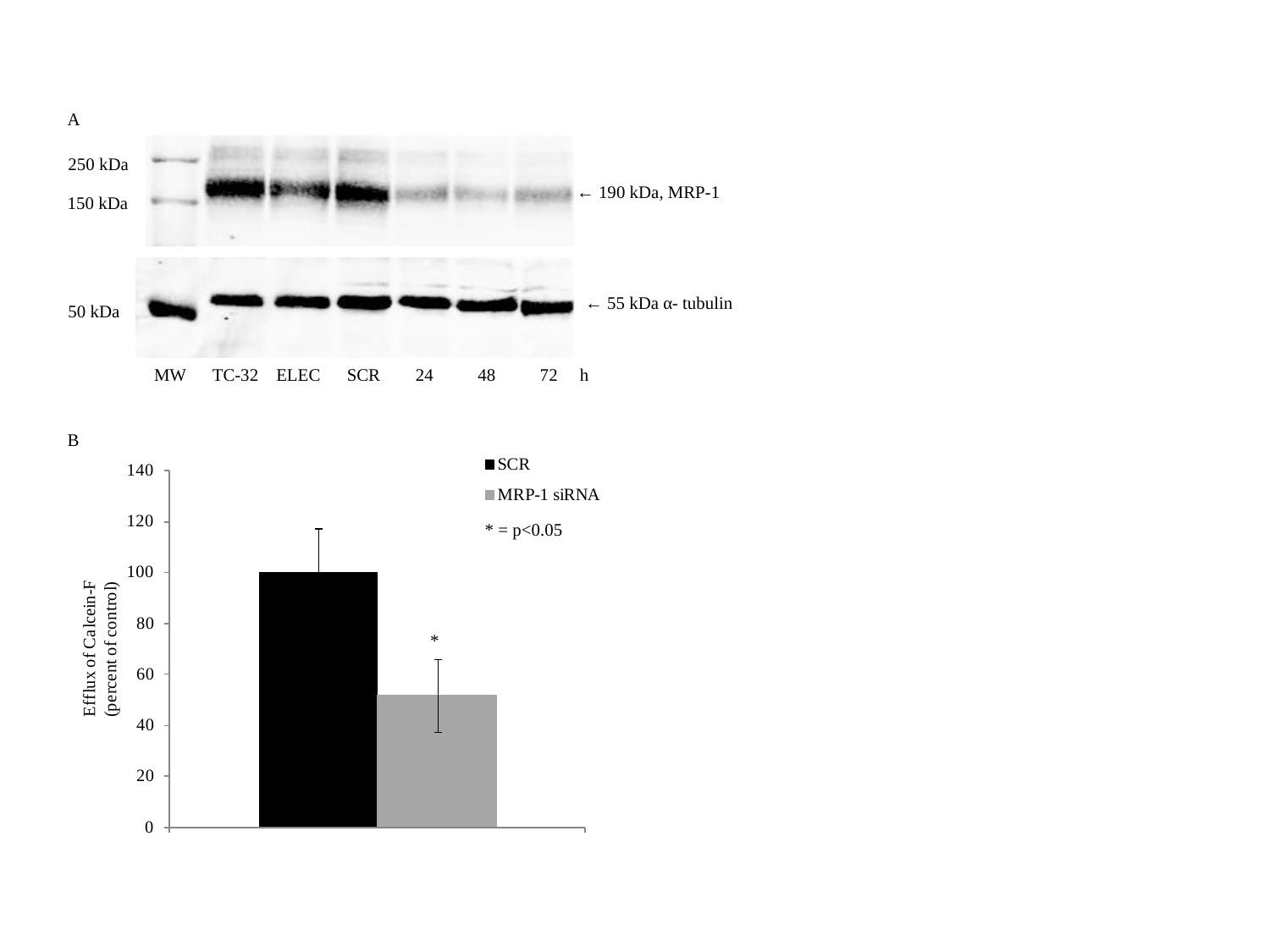

A
250 kDa
← 190 kDa, MRP-1
150 kDa
← 55 kDa α- tubulin
50 kDa
MW TC-32 ELEC SCR 24 48 72 h
* = p<0.05
*
B

Supplement: Supplementary Figure 4 [file bjc201240x4.ppt]

## Slide 1
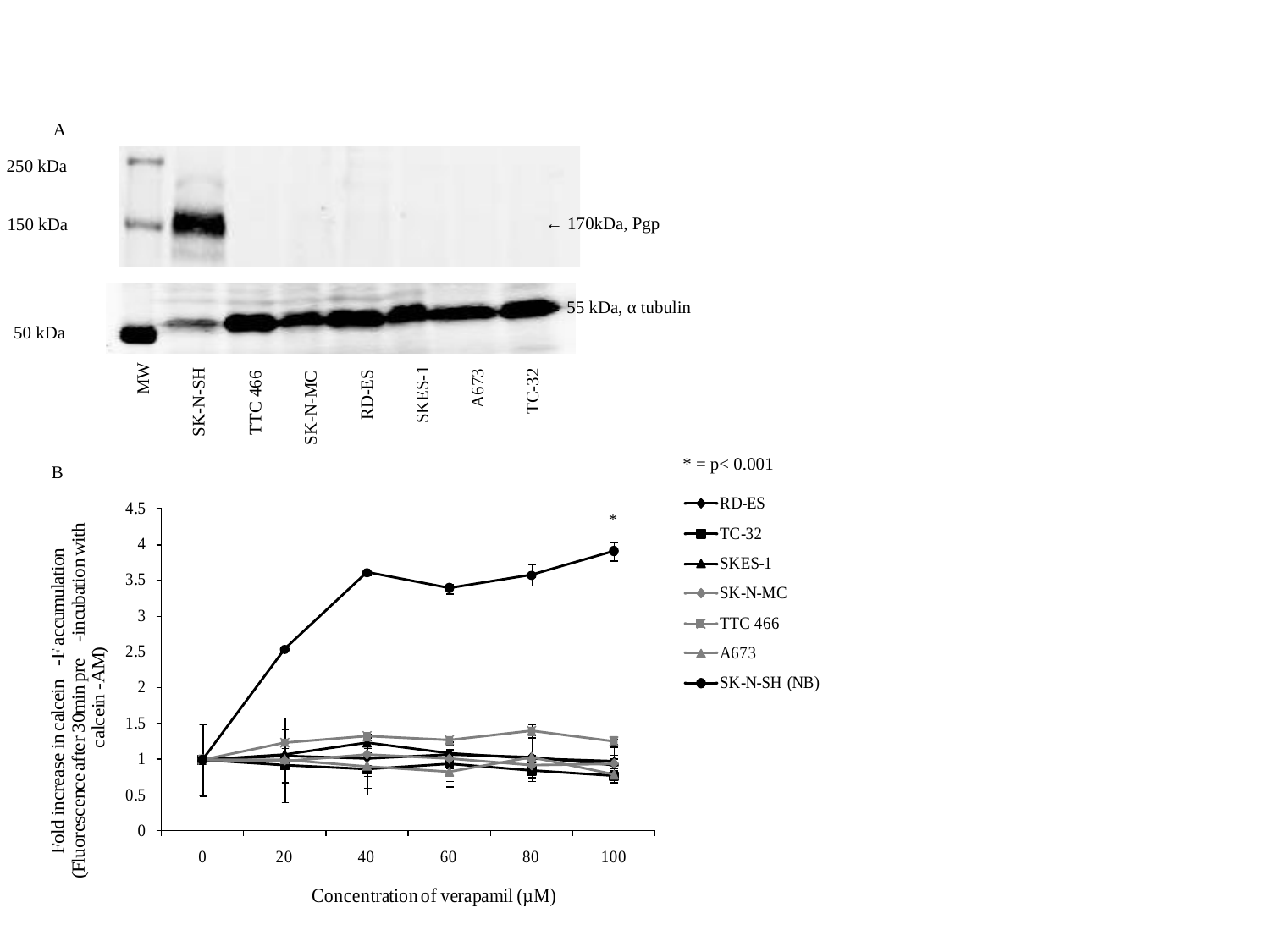

A
250 kDa
← 170kDa, Pgp
150 kDa
← 55 kDa, α tubulin
50 kDa
A673
MW
TC-32
RD-ES
SKES-1
SK-N-MC
TTC 466
SK-N-SH
* = p< 0.001
*
B

Supplement: Supplementary Figure 5 [file bjc201240x5.ppt]

## Slide 1
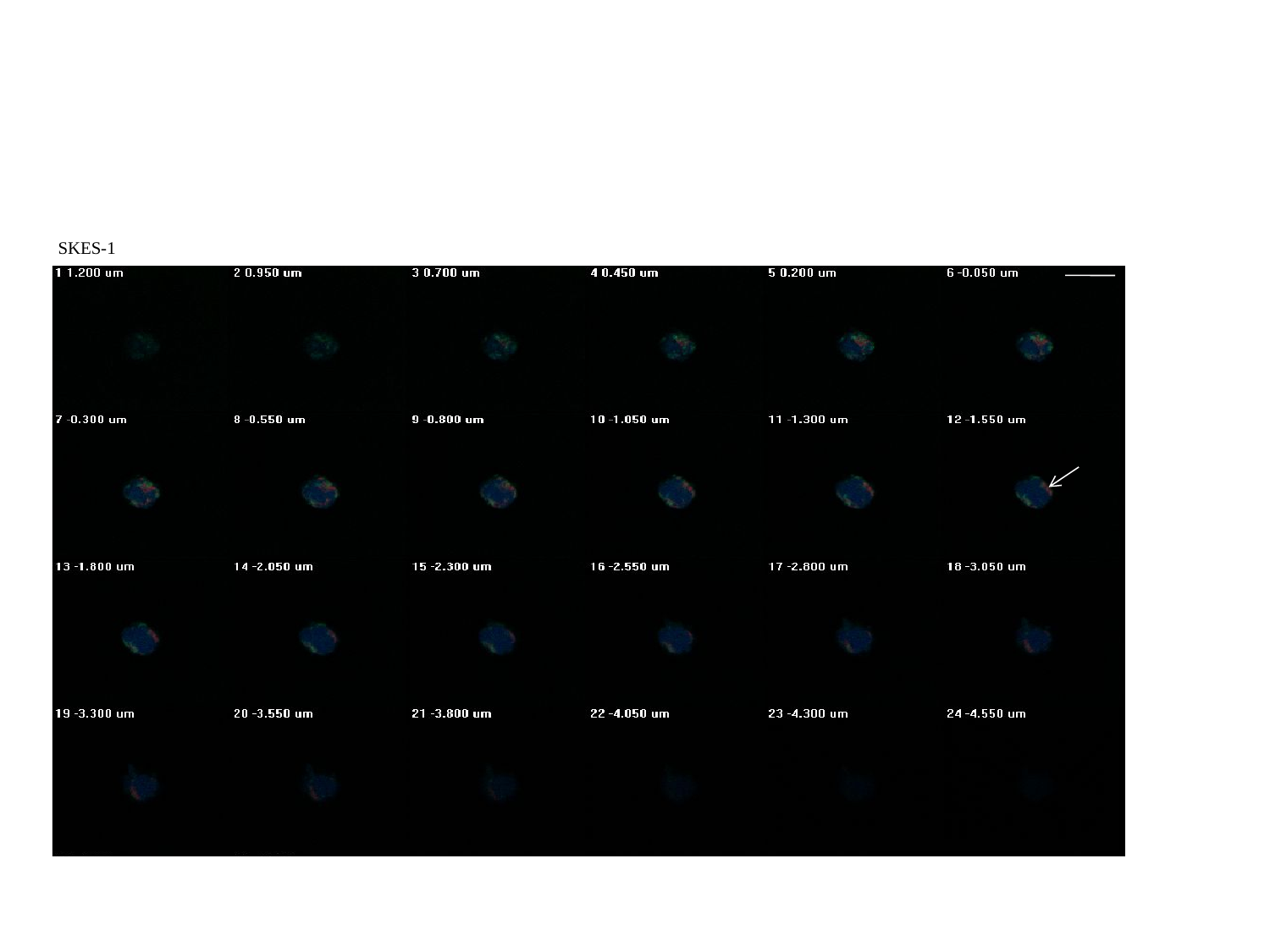

SKES-1

## Slide 2
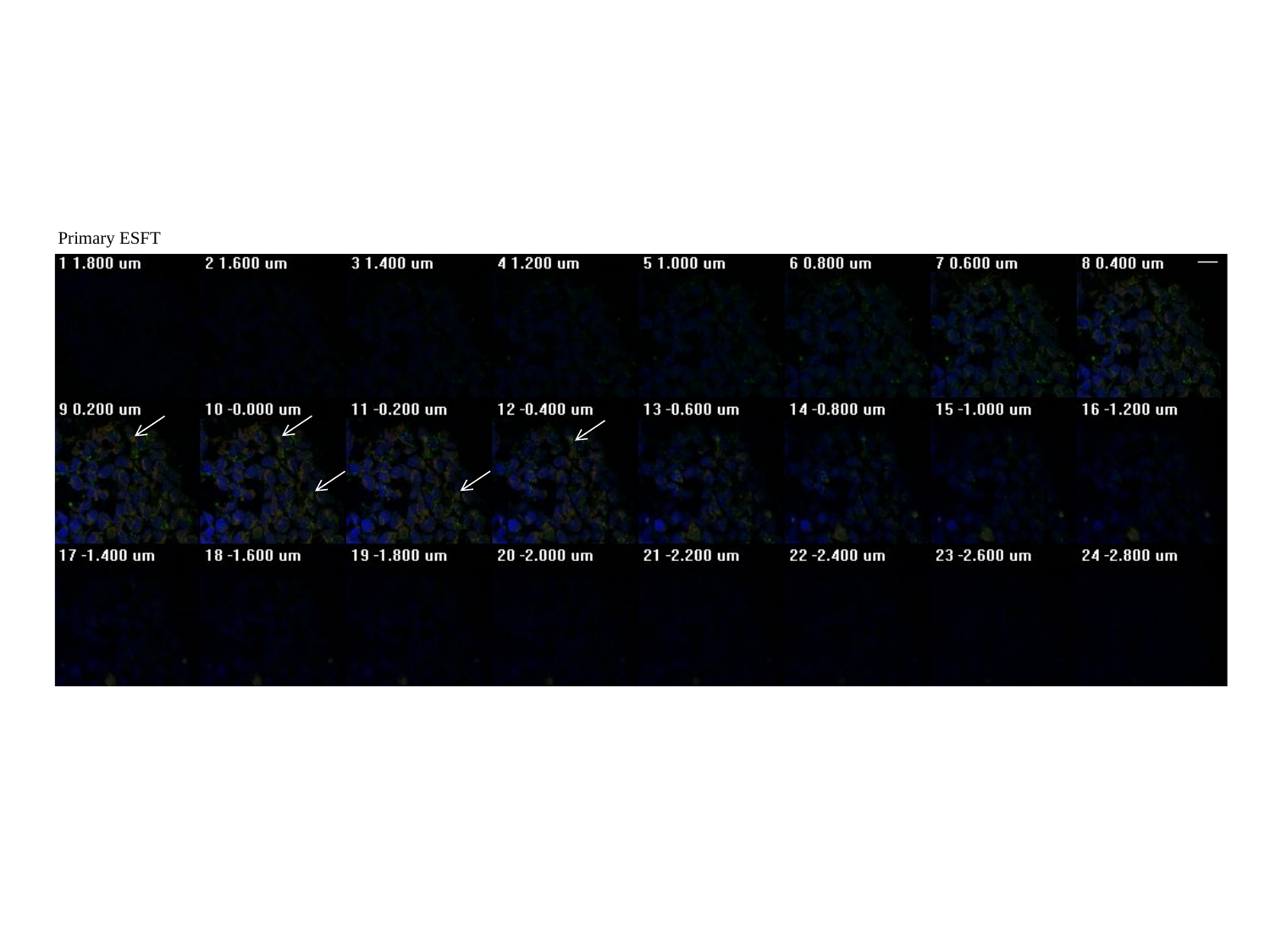

Primary ESFT

Supplement: Supplementary Figure 6 [file bjc201240x6.ppt]

## Slide 1
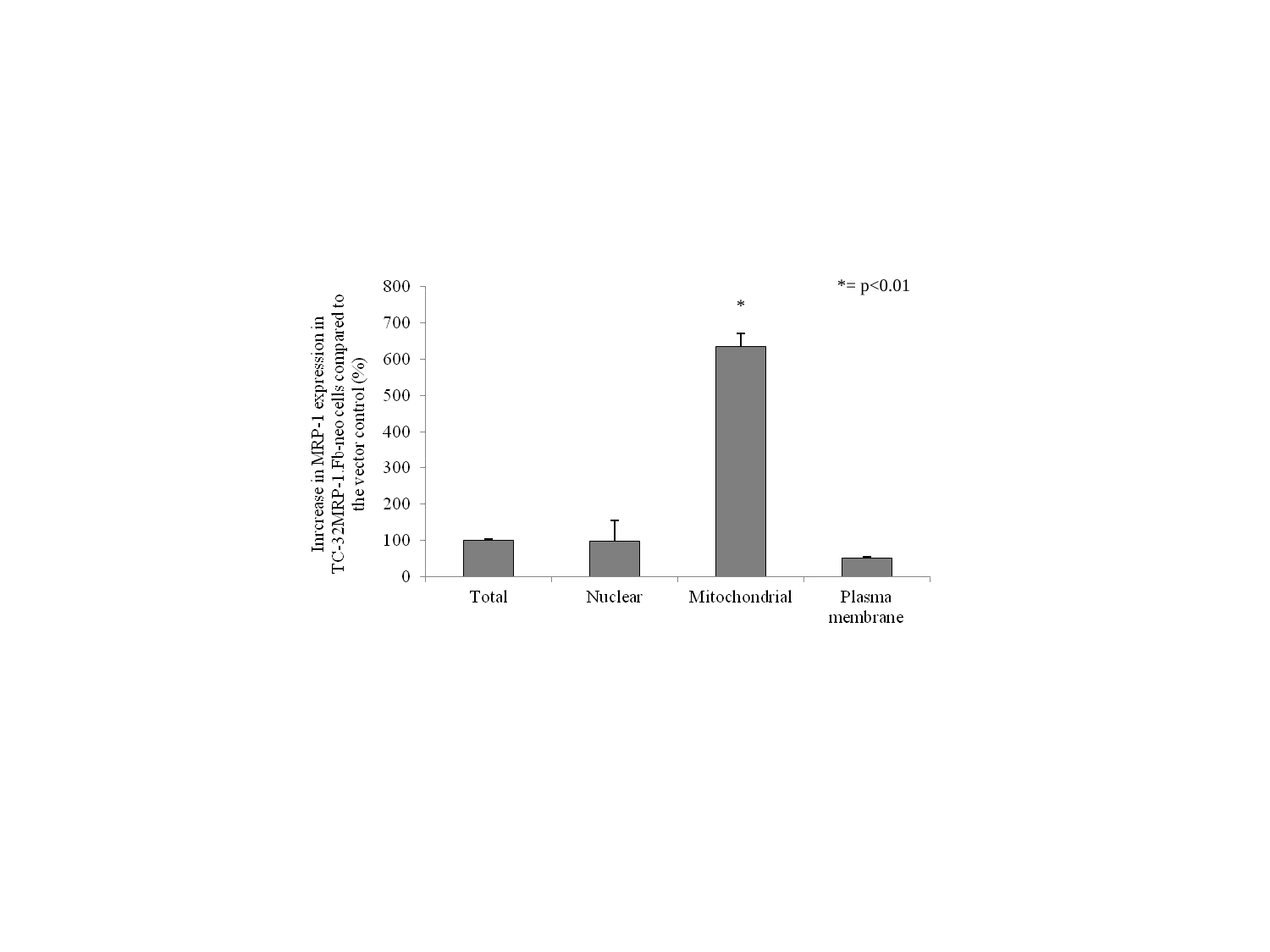

*= p<0.01
*

Supplement: Supplementary Figure 8 [file bjc201240x8.ppt]
