# Supplementary material for: Detection and characterisation of multi-drug resistance protein 1 (MRP-1) in human mitochondria
Source: Br J Cancer. 2012 Feb 21;106(6):1224–33. doi: 10.1038/bjc.2012.40 (PMC3304412; doi:10.1038/bjc.2012.40)
Supplement: Supplementary Figure 2 [file bjc201240x2.ppt]

## Slide 1
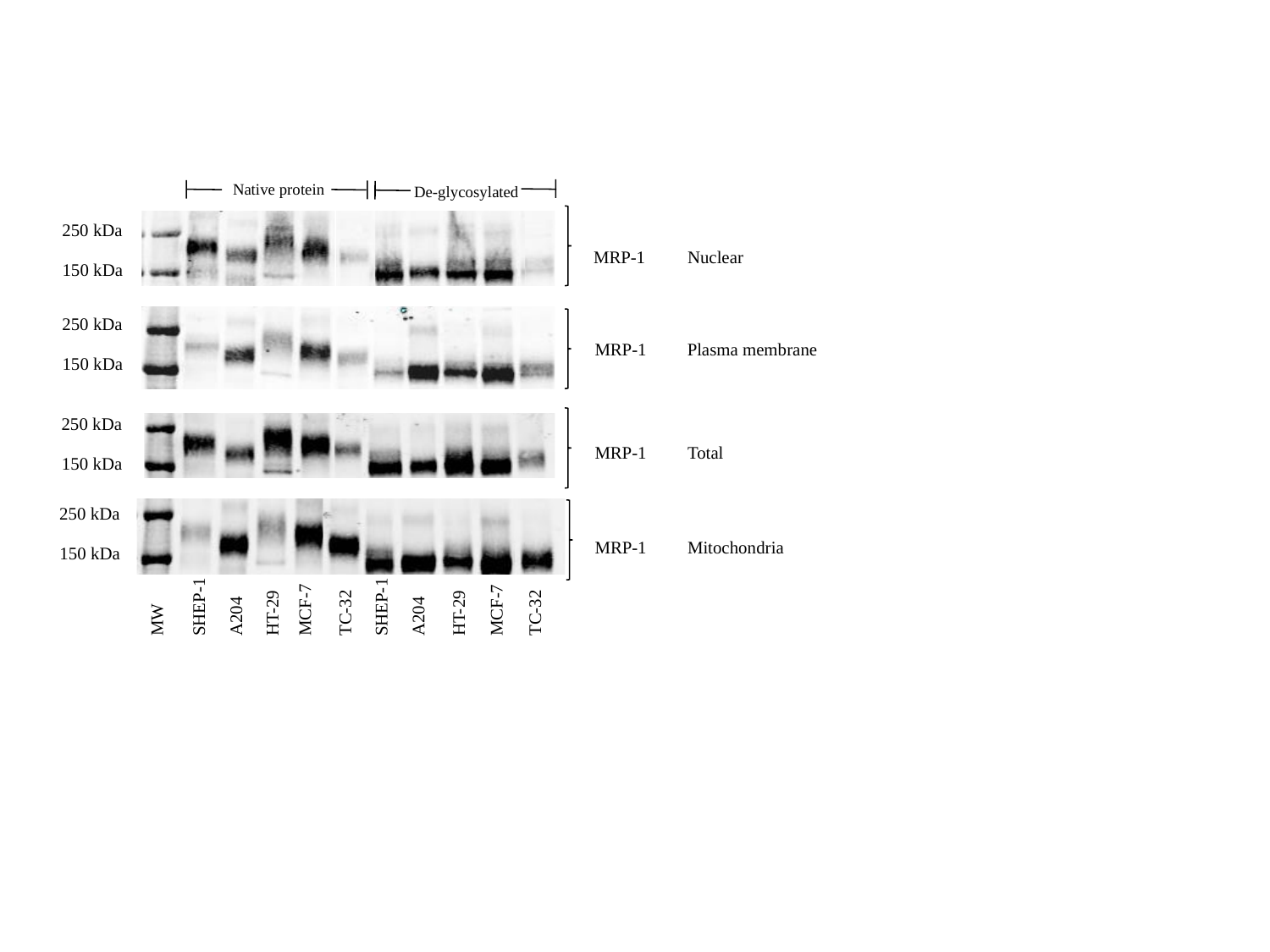

Native protein
De-glycosylated
250 kDa
MRP-1
Nuclear
150 kDa
250 kDa
MRP-1
150 kDa
Plasma membrane
250 kDa
MRP-1
150 kDa
Total
250 kDa
150 kDa
MRP-1
Mitochondria
TC-32
TC-32
SHEP-1
SHEP-1
A204
HT-29
MCF-7
MW
A204
HT-29
MCF-7
