# Supplementary material for: Detection and characterisation of multi-drug resistance protein 1 (MRP-1) in human mitochondria
Source: Br J Cancer. 2012 Feb 21;106(6):1224–33. doi: 10.1038/bjc.2012.40 (PMC3304412; doi:10.1038/bjc.2012.40)
Supplement: Supplementary Figure 7 [file bjc201240x7.ppt]

## Slide 1
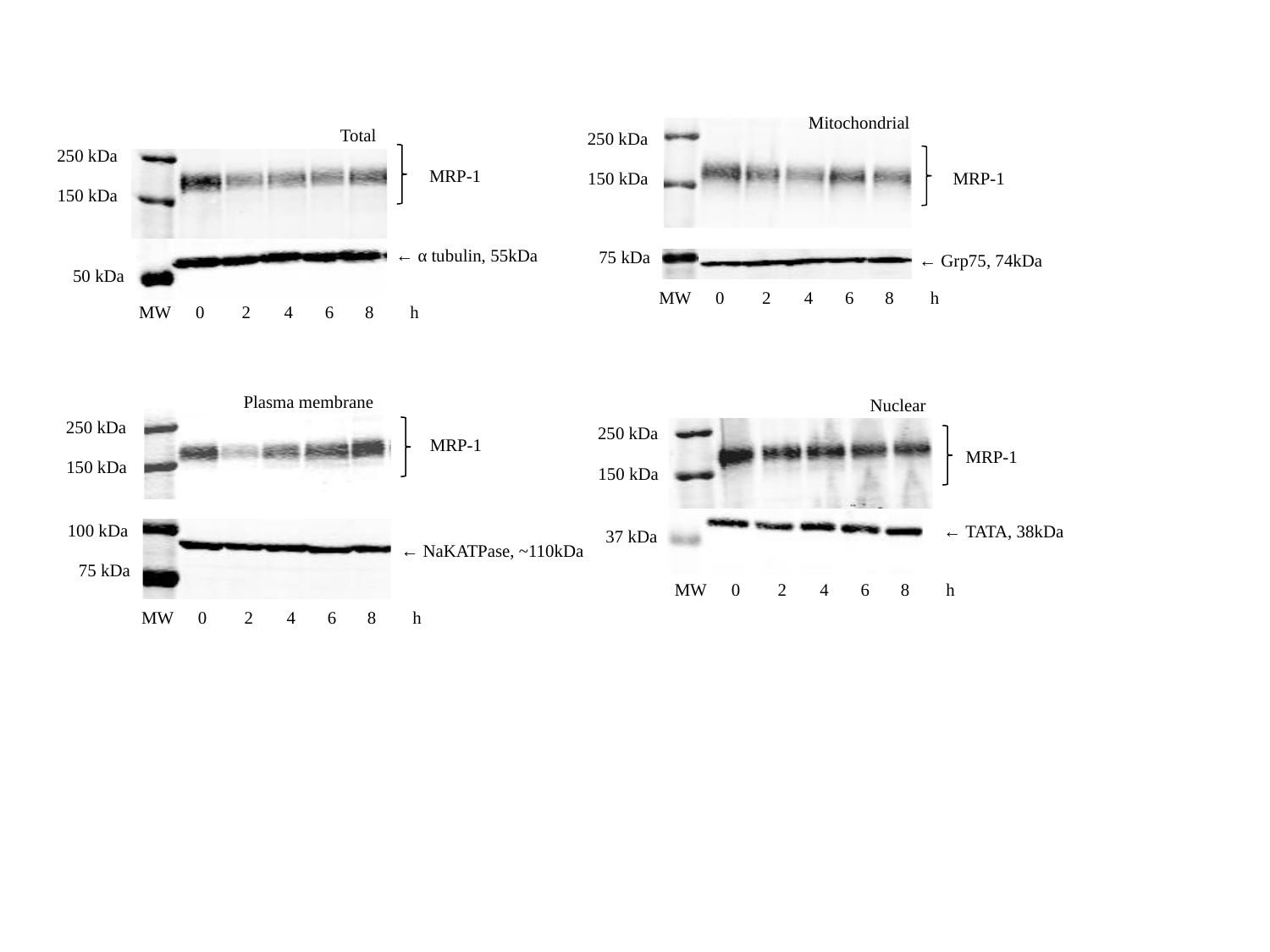

Mitochondrial
Total
250 kDa
250 kDa
MRP-1
MRP-1
150 kDa
150 kDa
← α tubulin, 55kDa
75 kDa
← Grp75, 74kDa
50 kDa
MW
0
2
4
6
8
h
MW
0
2
4
6
8
h
Plasma membrane
Nuclear
250 kDa
250 kDa
MRP-1
MRP-1
150 kDa
150 kDa
100 kDa
← TATA, 38kDa
37 kDa
← NaKATPase, ~110kDa
75 kDa
MW
0
2
4
6
8
h
MW
0
2
4
6
8
h
