# Supplementary material for: Detection and characterisation of multi-drug resistance protein 1 (MRP-1) in human mitochondria
Source: Br J Cancer. 2012 Feb 21;106(6):1224–33. doi: 10.1038/bjc.2012.40 (PMC3304412; doi:10.1038/bjc.2012.40)
Supplement: Supplementary Figure Legends [file bjc201240x9.doc]

**Supplementary Figure Legends**

(1) Inhibition of MRP-1 and Pgp activity in TC-32 and SK-N-SH cells using the inhibitors MK571 and verapamil respectively. Values represent the ratio of calcein-F fluorescence, compared to the initial fluorescence value. Results of three independent experiments (±SEM) (n=9). Inhibition of MRP-1 by MK571 in Pgp expressing SK-N-SH cells had no effect on calcein-F accumulation (MK571 does not inhibit Pgp activity). In addition, inhibition of Pgp by verapamil in TC-32 cells had no effect on calcein-F accumulation (reflecting the absence of Pgp expression). However, inhibition of Pgp by verapamil in the Pgp expressing SK-N-SH cells increased calcein-F accumulation. Inhibition of MRP-1 by MK571 in TC-32 cells had no effect on calcein-F accumulation but decreased the efflux of calcein-F over 60min (reflecting the inhibition of MRP-1 activity by MK571).

(2) MRP-1 protein expression in the subcellular fractions of cancer cells in native and de-glycosylated protein extracts prepared by differential centrifugation and determined by Western blot. MW=molecular weight marker.

(3) MRP-1 protein expression in the subcellular fractions of SKES-1 cells. Protein extracts prepared by differential centrifugation and expression of MRP-1 and the mitochondrial marker cytochrome C and the plasma membrane marker NaKATPase were determined by Western blot. MW=molecular weight marker.

(4) (A) Expression of MRP-1 protein in whole TC-32 cells by Western blot 24-72h post-electroporation with 400nM MRP-1 siRNA. Protein extracts from TC-32 cells electroporated (ELEC) and electroporated with a scrambled siRNA (400nM; SCR) were harvested at the final time point and included as negative controls. Expression of MRP-1 was determined by Western blot. α-tubulin was used to confirm equal loading of protein. MW=molecular weight marker; Electroporation alone or treatment with scrambled control siRNA had no effect on the expression of MRP-1.

(B) Activity of MRP-1 in whole SK-N-MC cells 48h post-electroporation with 400nM MRP-1 siRNA by the calcein-AM functional assay. Cells electroporated with a scrambled siRNA (400nM; SCR) were analysed as a negative control. Knockdown of MRP-1 expression decreased MRP-1 efflux activity by 49% (p<0.05) compared to cells electroporated with a scrambled siRNA (400nM; SCR).

(5) Pgp (A) protein expression and (B) activity in ESFT cell lines. Pgp expression and activity were determined by Western blotting and the calcein-AM functional assay respectively. SK-N-SH NB cells were used as a positive control for Pgp expression and activity. There is no Pgp expression or activity in ESFT cell lines.

(6) Full Z-stack images of an SKES-1 cell and a region of a primary ESFT (same sample as Figure 2) created and rendered using the Nikon NIS-elements software. Arrows represent regions of co-localisation. Images are representative of the tissue population analysed. Scale bar = 10µm.

(7) Protein expression of MRP-1 in TC-32 cells after incubation with etoposide (7.2nM). Protein extracts from subcellular fractions were prepared and expression determined by western blot. α-tubulin, sodium potassium ATPase, Grp75 and TATA TBP were used to check equal loading of total, plasma membrane, mitochondrial and nuclear fractions respectively. Results are representative of 3 independent sets of extracts. MW=molecular weight marker.

(8) Percentage increase in MRP-1 expression in the TC-32MRP-1.Fb-neo cell fractions compared to the vector control (%). Mitochondrial MRP-1 increased preferentially compared to other cell fractions (p<0.01). The intensity of a band visualised on the membrane was quantified by densitometry using the Li-cor Odyssey infrared imaging system software (Li-cor Biosciences). Protein expression was expressed as a ratio of densitometry value of MRP-1 relative to the densitometry value of the corresponding loading control protein band. Results are shown as mean ±SEM of four independent Western blots.
